# Seroprevalence and Risk Factors for *Taenia solium* Cysticercosis in Rural Pigs of Northern Peru

**DOI:** 10.1371/journal.pntd.0001733

**Published:** 2012-07-17

**Authors:** César M. Jayashi, Gianfranco Arroyo, Marshall W. Lightowlers, Héctor H. García, Silvia Rodríguez, Armando E. Gonzalez

**Affiliations:** 1 The University of Melbourne, Werribee, Victoria, Australia; 2 Universidad Nacional Mayor de San Marcos, Lima, Peru; 3 Instituto Nacional de Ciencias Neurológicas, Lima, Peru; Universidad Nacional Autónoma de México, Mexico

## Abstract

*Taenia solium* is a cestode parasite that causes cysticercosis in both humans and pigs. A serological survey was undertaken to assess the seroprevalence and risk factors associated with porcine cysticercosis in the rural district of Morropon, Peru. Pigs aged between 2 and 60 months were assessed by the Enzyme-linked Immunoelectrotransfer blot (EITB) assay to determine their serological status against porcine cysticercosis in a cross-sectional study. A total of 1,153 pigs were sampled. Porcine seroprevalence was 45.19% (42.31–48.06). The information about the animals and households was analyzed and risk factors associated with seroprevalence were determined by a multivariate logistic regression analysis. In the porcine population, the risk of being seropositive increased by 7% with every month of age (OR 1.07, 95% CI 1.05–1.09), and by 148% for pigs living in East Morropon (OR 2.48, 95% CI 1.82–3.37). Whereas, the presence of latrines in a household decreased the risk of being seropositive by 49% (OR 0.51; 95% CI 0.39–0.67). Sex and rearing system did not represent either risk or protective factors associated with the seroprevalence of porcine cysticercosis. The findings of this study could be used for further development of control programs that might focus on similar population groups within rural communities of developing countries where cysticercosis is endemic.

## Author Summary

*Taenia solium* causes taeniasis in humans and cysticercosis in humans and pigs. In humans the parasite may infect the central nervous system and cause neurocysticercosis. The World Health Organization (WHO) estimated that over 50,000 deaths per year are due to neurocysticercosis and the disease is also the main cause of acquired epilepsy. Pigs act as intermediate hosts for the parasite's transmission. Porcine cysticercosis causes economic losses to farmers of developing countries, because infected pork has reduced value or may be condemned. Previous studies have identified risk factors for *T. solium* infection in pigs in various parts of the world; however, findings are contradictory or not consistent. In this study, particular areas in which pigs lived and age (older pigs were at higher risk) increased the risk of being seropositive; whereas the use of latrines decreased their risk of being seropositive. The results of this study contribute to epidemiology of porcine cysticercosis in rural areas, which is relevant for establishing effective control programs in rural locations with similar characteristics.

## Introduction

Neurocysticercosis is a disease that affects humans mainly in developing countries, causing serious morbidity and mortality [1]. *T. solium* infection in pigs causes production losses to farmers because infected meat has reduced value or may be condemned at slaughterhouses. In rural areas infected pig carcasses can be sold avoiding the legitimate commercial distribution [2]. Epilepsy caused by neurocysticercosis in humans incurs many economic and social costs. It affects workers within highly productive age groups reducing work productivity [3]. Stigmatization arises as a serious problem for the farmers/villagers with neurocysticercosis since they are relegated and do not have the benefit of being part of the normal community life [4], [5]. In Peru, epidemiological studies based on serological surveys using the Enzyme-linked Immunoelectrotransfer blot (EITB) have determined variable porcine cysticercosis seroprevalences in the three natural regions the country: coast, highlands and Amazon. The EITB test has been commonly used to determine the epidemiological characteristics of the taeniasis/cysticercosis complex [6]. Studies done in the Peruvian Amazon found seroprevalences of porcine cysticercosis that ranged from 28% to 49% [6]. In the Peruvian Highlands, a region with a high poverty rate, the disease is known to be hyper-endemic with seroprevalences up to 75% [1]. Studies in the Northern Coast of Peru found seroprevalences that ranged from 13% [4] to 30.8% [7].

Few studies on the risk factors for porcine or human cysticercosis in Peru have been done [7], [8]. These studies assessed the factors in the human and pig populations that are associated with the seroprevalence of porcine cysticercosis in rural villages of the Highlands and Coast of Peru. However, some social, economic, geographic and environmental characteristics are specific to particular locations and therefore risk factors may differ from communities located in different regions. A cross-sectional serological survey in pigs was undertaken to determine the seroprevalence of porcine cysticercosis and identify the risk factors for *T. solium* transmission. The survey was conducted in 14 villages located in the district of Morropon, Piura, Peru using the EITB as the diagnostic test. The particular region investigated was selected at the beginning based on limited, anecdotal knowledge which suggested a high rate of *T. solium* transmission in the region.

## Materials and Methods

### Ethics Statement

The study complied with the “National Health and Medical Research Council Australian Code of Practice for the Care and Use of Animals for Scientific Purposes” (7^th^ edition, 2004) ethics standard. The study protocol was approved by the scientific boards at the Veterinary Faculty, the University of Melbourne, Australia and at the Veterinary Faculty, San Marcos National University, Peru. Study permissions were obtained from the Municipality of Morropon, from village leaders and from the pig owners. Due to a high level of illiteracy among villagers, the scientific board at the Veterinary Faculty, San Marcos National University approved the use of oral consent. Oral consent was obtained from household owners prior to them providing answers to the questionnaire and using their animals in the study; consent was recorded through the completion of the questionnaire.

### Study Area

Morropon is a province in the department of Piura, Peru. It is located in Northeast Peru, 82.3 km from Piura, the closest major city. The district of Morropon is the commercial center of the region and villages are located in the surroundings. The altitude is 131 meters above sea level. The climate is dry and hot from May to December, with heavy rain fall from January to April.

### Sample Size

The sample size required for the study was obtained through the Sample Size formula for estimation of a proportion for infinite populations [9]. The referential prevalence used was 26% [10] and a 95% confidence level. The minimum number of animals calculated by the sample size formula was 296. This number of animals was required in order to represent a valid sample of the total population; however, due to the availability of resources and to not affect the villager's compliance by selecting some houses only, it was decided to undertake a census sampling, attempting to include every pig from all the villages in the study.

### Diagnostic Criteria: Enzyme-Linked Immunoelectrotransfer Blot

Sera samples for processing were sent to the diagnostic laboratory of the Instituto Nacional de Ciencias Neurológicas (Lima, Peru) to perform the EITB test. The EITB test used the same methodology described by Tsang et al. [11] and Gonzalez et al. [12]. The EITB assay for diagnosis of human or porcine cysticercosis identifies as being positive any sample having reactivity with any one of seven lentil-lectin, affinity-purified *Taenia solium* metacestode glycoprotein antigens (GP50, GP42-39, GP24, GP21, GP18, GP14 and GP13). The sensitivity and specificity of this assay in pigs were reported to be 100% [12].The sensitivity of this assay in humans is 98% and its specificity reaches 100% [13].

### Serological Survey

A serological survey of pigs was undertaken in the following villages: Alto Mambluque, Bocanegra, Coca, El Chorro, Faical, Franco, Franco Alto, Franco Bajo, La Bocana, Mambluque, Maray, San Francisco, Talanquera, and Zapotal located in the district of Morropon. These villages were selected based on various characteristics, such as socio-economic, accessibility, recent cases of human or porcine cysticercosis and acceptance and enthusiasm from villagers and village authorities. Pregnant sows and animals younger than 2 months were not included in the survey. All animals were ear-tagged to identify the animals and their blood samples. Animals were also vaccinated against Classical Swine Fever (CSF) as an incentive to the livestock owners to participate in the survey.

### Household Questionnaire

The household owners were interviewed and information about the household, living conditions and pig husbandry practices were recorded. The respondent was the owner of the household (husband or wife or any adults living in the household). Their oral consent was obtained prior to answering the questionnaire and taking blood samples from the pigs. The questionnaire was concurrently conducted while blood samples of pigs were taken. Specific information was recorded for each animal including: identification number, age and sex. Information about the households included: the presence of latrines, the rearing-system used and the village to which households belonged. The data was analyzed anonymously. Each household's data was kept confidential and not shared with any other household.

### Statistical Analyses

Data was entered on Microsoft Office Excel 2007 datasheets (Microsoft). Statistical calculations were performed using the computer program STATA 10.0 (StataCorp LP, USA). Descriptive analyses were based on frequencies and percentages for qualitative variables, and means with their confidence intervals for quantitative variables. Bivariate analyses were performed calculating odds ratios (OR) to assess the variables: sex, age, village, rearing system, and presence/absence of a latrine as potential risk factors.

**Figure 1:**
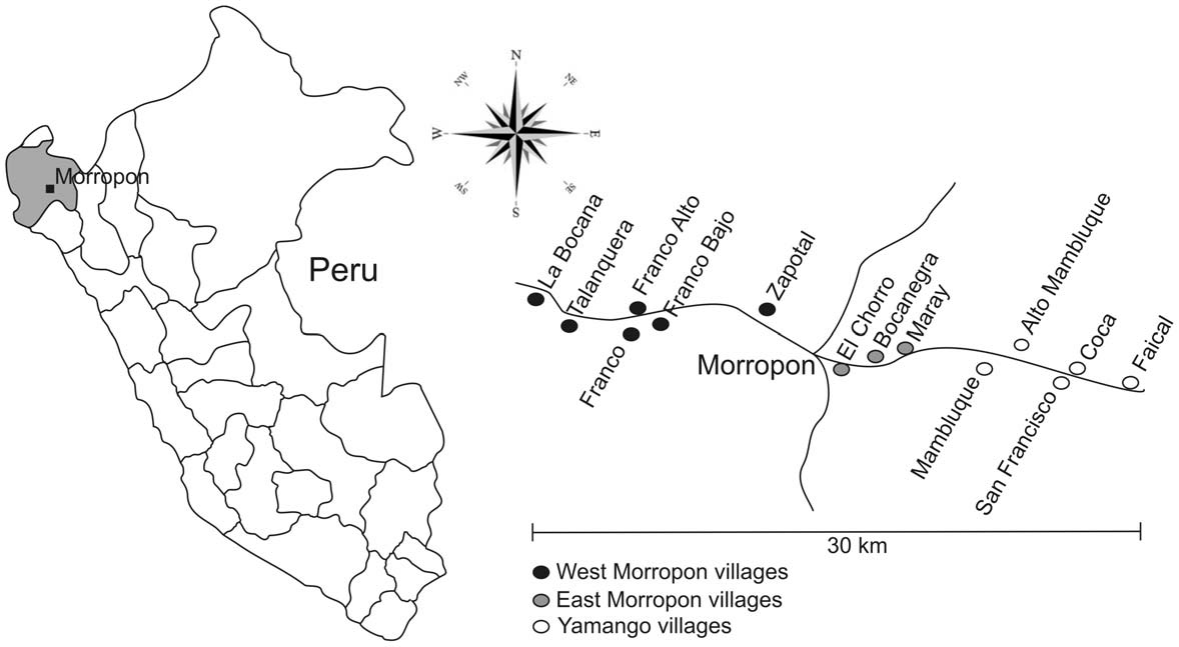
Study area and village distribution.

For practical and statistical purposes, Morropon villages were assigned into three different areas: East Morropon, West Morropon and Yamango. The two criteria used to divide the area were geographical and road access. Yamango area included Faical, Coca, San Francisco, Mambluque and Alto Mambluque villages. East Morropon included Maray, El Chorro and Bocanegra villages. West Morropon included Zapotal, Franco, Franco Alto, Franco Bajo, Talanquera and La Bocana villages. Figure 1 shows a schematic map of the study area.

The aggregated effect of the sex, age (months), area, rearing system and presence/absence of a latrine over the binary dependent variable, EITB result, was modeled by a multivariate logistic regression analysis. The multivariate model included variables of epidemiological and biological interest. Adjusted relative risks for pig seropositivity to cysticercosis with the corresponding 95% confidence interval (95% CI) and *p* values were estimated by a multivariate logistic regression. A *p* value of less than 0.05 was considered to indicate statistical significance.

## Results

### Descriptive Analysis: Seroprevalence

The number of animals sampled, the total seroprevalence and the seroprevalence per village are shown in Table 1. A total of 1153 animals were sampled belonging to 306 households. The population sample represented approximately 90% of the total pig population in the study villages. Seropositive animals to the EITB test were found in all 14 villages of study. From the 1153 sampled animals, 521 were positive, giving a seroprevalence of 45.19% (42.31–48.06). The seroprevalences per village ranged from 14.5% for Franco Alto to 81.2% for Bocanegra. When analyzed by area, East Morropon had the highest porcine cysticercosis seroprevalence, 56.6% (176/311).

**Table 1:** Seroprevalence of porcine cysticercosis in the villages of Morropon determined by EITB.

	EITB result	
Village	Positive/total	Seroprevalence (%) ± (95% CI)
Coca	83/165	50.30 (42.43–58.17)
Faical	22/62	35.48 (23.74–48.66)
San Francisco	45/69	65.21 (52.79–76.29)
Mambluque	13/33	39.39 (22.91–57.86)
Alto Mambluque	10/32	31.25 (16.12–50.00)
Maray	83/184	45.11 (37.77–52.59)
El Chorro	57/83	68.67 (57.56–78.41)
Bocanegra	36/44	81.82 (67.29–91.81)
La Bocana	54/101	54.46 (43.27–63.45)
Talanquera	55/149	36.91 (29.16–45.19)
Franco	22/77	28.57 (18.85–40.00)
Franco Alto	9/62	14.52 (6.85–25.78)
Franco Bajo	10/37	27.03 (13.79–44.12)
Zapotal	22/55	40.00 (27.02–54.10)
**Total**	521/1153	45.19 (42.31–48.06)

### Descriptive Analysis: Population Characteristics

The West Morropon area had the highest number of sampled pigs, 41.7% (481/1153), while East Morropon area had the smallest number of sampled animals, 27.0% (311/1153). The mean age of the population was 8.5±8.3 months, with ages ranging from 2–60 months. To have a gross overview of the age of the study population, age was arranged as an ordinal variable using ≤8, 9–16 and >16 months as reference ages (for the multivariate analysis age was modeled as a continuous variable). There was a higher proportion of “young” animals, with pig ≤8 months representing 70.8% (816/1153) of the total sample, pigs between 9–16 months represented 18.6% (214/1153) of the population, and pigs ≥16 months represented 10.7% (123/1153). There were slightly more female pigs, 55.5% (640/1153) in the population compared to males, 44.5% (513/1153). The majority of pigs were reared using the free-roaming system, 56.3% (649/1153) in contrast to pigs reared in confined conditions, 43.7% (504/1153). The seroprevalences in animals reared confined and free-roaming were 44.8% (226/504) and 45.5% (295/649) respectively. From the 306 households that participated in the serological survey, 43.1% (132/306) did not have latrines, and 42.5% (490/1153) of the sampled animals belonged to households without latrines. All interviewed families (306) knew about the occurrence of porcine cysticercosis in the area of study (known as “triquina” by the locals). However, less than 1% (2/306) associated the pig habit of coprophagy with the onset of the disease in the animals (data not shown). A summary of the various exposure and biological variables and the seroprevalence (EITB result) is presented in Table 2.

**Table 2:** Porcine seroprevalence arranged by biological and exposure variables determined by EITB.

	EITB result	
Variables	Positive/total	Seroprevalence (%) ± (95% CI)
**Age**		
≤8 months	291/816	35.66 (32.37–38.95)
9–16 months	145/214	67.76 (61.47–74.04)
>16 months	85/123	69.11 (60.90–77.31)
**Area**		
Yamango	173/361	47.92 (42.66–53.21)
East Morropon	176/311	56.59 (50.88–62.17)
West Morropon	172/481	35.76 (31.47–40.22)
**Sex**		
Female	305/640	26.54 (18.60–26.12)
Male	216/513	22.36 (22.90–30.17)
**Rearing system**		
Confined	226/504	44.84 (40.49–49.19)
Free roaming	295/649	45.45 (41.61–49.29)
**Presence of latrines**		
Yes	252/633	38.01 (34.31–41.71)
No	269/490	54.90 (50.48–59.31)
**Total**	521/1153	

### Multivariate Analysis

A multivariate logistic regression analysis was performed for all variables included in the study with the exception of the variable “village”. Instead of “village”, the variable “area” (the three areas into which Morropon district was divided) was included in the logistic regression model. The variable age (months) was analyzed as a continuous variable. The odds ratios with confidence intervals as well as the *p* values of a logistic regression to determine the risk factors for the EITB seropositivity are shown in Table 3. The analysis determined that the pig's age was a risk factor for reaction against the EITB test in seropositive animals (adjusted by sex, area, rearing method and presence/absence of latrine). The odds of being seropositive increased by 7% with every month of age in our study population (*p*<0.01). The multivariate analysis also determined that animals belonging to the villages in the East Morropon had a 148% increased risk of being seropositive compared to animals from West Morropon. The presence of latrines in households was a protective factor for the occurrence of being seropositive, finding a 49% decreased risk in houses with latrines versus houses without them (*p*<0.01). Finally, variables such as sex and rearing system did not represent either risk or protective factors associated with the seroprevalence of porcine cysticercosis.

**Table 3:** Effect of multiple variables on the EITB assay results expressed as odds ratios (OR).

Variables	Odds ratio (OR)	*p* value	95% CI
Age (months)*	1.07	<0.001‡	1.05–1.09
Sex	0.95	0.720	0.74–1.23
Yamango area†	1.34	0.079	0.96–1.87
East Morropon area†	2.48	<0.001‡	1.82–3.37
Free-roaming	0.94	0.994	0.71–1.24
Presence of latrines	0.51	0.001‡	0.39–0.67

*: : Age was modeled as a continuous variable (Age range: minimum = 2, maximum = 60).

**†:** : West Morropon area used as baseline.

**‡:** : Significantly different.

## Discussion

This study found that the age of a pig and the area where the pig lived increased the risk of being seropositive to *T. solium* whereas the presence of latrines was found to decrease the risk of being seropositive. The seroprevalence of porcine cysticercosis was found to be 45.19% which is similar to the seroprevalence of porcine cysticercosis found in areas where the disease is considered hyper-endemic [1].

The finding that the presence of latrines was a protective factor to decrease the seroprevalence of porcine cysticercosis is not surprising as the use of latrines has been proposed to control cysticercosis worldwide by multiple authors [14], [15] and has been reported to be a protective factor for the occurrence of the disease [16], [17], [18], [19]. However, in other studies it was not associated with the occurrence of porcine cysticercosis [18] and in some cases it acted as a risk factor [20]. In our study, pigs were seen feeding on human feces near latrines. The access of pigs to human feces has been shown to be a risk factor for porcine cysticercosis [21]. Based on our observations, it appears that the presence or absence of latrines is as important as the knowledge required to use them properly.

In Morropon, an increase in the pig's age was a risk factor for being seropositive, which agrees with studies from Cameroon, Mozambique and Mexico [7], [22], [23]. However, Ngowi et al. [18] reported that the age was not a risk factor for the occurrence of porcine cysticercosis. It has been described that older animals have a higher chance of accessing human feces since younger animals have a disadvantage when foraging and scavenging [19]. Adult pigs also have a higher frequency of feces consumption compared to piglets [24]. In our study, the seroprevalences observed in young pigs may be also a reflection of the transfer of maternal antibodies [25]. Because of the association between seroprevalence and pig's age in rural communities, the effectiveness of control programs could be affected by the presence of older animals, as they might be reservoirs for the disease.

Peruvian rural families commonly use the pig as the equivalent of a savings account [26]. Pigs act as recyclers of waste thrown on the streets and walking paths, including feces. There is evidence that indicates that restraining pigs, which provides varying confinement (different locations and tethering length), may reduce the seroprevalence of porcine cysticercosis in rural areas [16], [27]. It has been reported elsewhere that using a free-range husbandry system increases the risk of acquiring cysticercosis [21], [23]. However, in this study the free-range rearing system was not a risk factor for porcine cysticercosis seroprevalence and could be explained by 1) the presence of human feces in the pig pens (due to open-air defecation); 2) the contamination of the environment by the presence of chickens in the households where pigs were raised (Jayashi, personal observations) since their feeding behavior promotes the spreading of *Taenia spp*. eggs [28]; and, 3) the inadequate construction of the pens that allows pigs to often “escape” their confinement and have access to human waste.

The villages in the area of East Morropon area are closer to the district center and have less harvesting areas. These properties had less land and animals and owners could be considered poorer than people living in villages at longer distances from the district center. Under these conditions the source of food is scarce and pigs are forced to scavenge for food and may more frequently ingest human feces, which could explain why pigs living in this area had increase odds for being seropositive.

This study had some limitations. Studies that have relevant information about the prevalence of porcine cysticercosis including our study are based on EITB serology rather than definitive *post mortem* examination [1], [4], [6], [20]. EITB has been widely used for epidemiological studies following Gonzalez's et al. [12] results, in which EITB was determined as being 100% specific and 100% sensitive in pigs. Based on these findings, EITB was considered as the gold standard test to diagnose porcine cysticercosis. However, some evidence shows that EITB serology does not correlate perfectly to necropsy results and in many cases animals are seropositive and necropsy is negative [10], [29]. It has been suggested that a positive serology result and a negative necropsy result is due to exposure of the animal to *T. solium* caused by an aborted infection which did not lead to mature cysticerci or detectable lesions [12]. A similar situation is presumed to exist with human serology [30]. Therefore, the risk of animal being seropositive in our study does not necessarily represent the risk of the animal being positive at necropsy.

This study describes the risks associated with the disease transmission in an area where porcine cysticercosis is highly prevalent. It expands the information available regarding the epidemiology of porcine cysticercosis in rural farming systems. The information obtained in this study was used for making the decision to proceed with a field vaccination trial in pigs to prevent cysticercosis in rural Peru. Nevertheless, this knowledge may also be used for further development of control programs that might focus in particular population groups within rural communities of developing countries where porcine cysticercosis and neurocysticercosis are endemic.
